# Associations between biomarkers at discharge and co-morbidities and risk of readmission after community-acquired pneumonia: a retrospective cohort study

**DOI:** 10.1007/s10096-018-3224-8

**Published:** 2018-03-29

**Authors:** Pelle Trier Petersen, Gertrud Baunbæk Egelund, Andreas Vestergaard Jensen, Stine Bang Andersen, Merete Frejstrup Pedersen, Gernot Rohde, Pernille Ravn

**Affiliations:** 1 Department of Pulmonary and Infectious Diseases, Nordsjællands Hospital, Dyrehavevej 29, 3400 Hillerød, Denmark; 2 University of Copenhagen, Copenhagen, Denmark; 3 Department of Clinical Biochemistry, Nordsjællands Hospital, Hillerød, Denmark; 4 Department of Respiratory Medicine, Medical Clinic I, Goethe University Hospital, Frankfurt/Main, Germany; 5 CAPNETZ STIFTUNG, Hannover Medical School, Hannover, Germany; 6 Medical Department O, Herlev and Gentofte Hospital, Gentofte, Denmark

**Keywords:** Community-acquired pneumonia, Readmission, Risk factor, Biomarker

## Abstract

To investigate whether hemoglobin, white blood cell count (WBC), urea, sodium, albumin, and C-reactive protein at discharge in patients hospitalized for community-acquired pneumonia (CAP) are associated with 30-day readmission. This study is a retrospective cohort study, which included all adult patients discharged after hospitalization for CAP from three Danish hospitals between January 2011 and July 2012. The outcome was all-cause, unplanned, 30-day readmission. Biomarker concentrations at discharge were transformed into binary variables by using either upper or lower quartiles as cut-off; the upper quartile was used for WBC, urea, and C-reactive protein, and the lower quartile was used for hemoglobin, sodium, and albumin. The study population consisted of 1149 patients. One hundred eighty-four (16.0%) patients were readmitted. Independent risk factors of readmission were WBC ≥ 10.6 cells × 10^9^/L (hazard ratio 1.50; 95% CI, 1.07–2.11) and albumin <32 g/L (hazard ratio 1.78; 95% CI, 1.24–2.54) at discharge and the presence of ≥ 2 co-morbidities (hazard ratio 1.74; 95% CI, 1.15–2.64). When WBC, albumin, and co-morbidities were combined into a risk-stratification tool, there was a step-wise increase in risk of readmission for patients with 1, 2, or 3 risk factors with hazard ratios of 1.76 (95% CI, 1.25–2.49), 2.59 (95% CI, 1.71–3.93), and 6.15 (95% CI 3.33–11.38), respectively. WBC ≥ 10.6 cells × 10^9^/L and albumin < 32 g/L at discharge and the presence of ≥ 2 co-morbidities were independently associated with increased risk of 30-day readmission.

**Electronic supplementary material:**

The online version of this article (10.1007/s10096-018-3224-8) contains supplementary material, which is available to authorized users.

## Introduction

Community-acquired pneumonia (CAP) is a common cause of hospitalization, and with as many as 1 in 5 patients returning to the hospital within 30 days after discharge, readmission is a significant burden to both patients and healthcare systems [1–4].

Readmission may be prevented with post-discharge interventions, but success and cost-effectiveness partly depend on accurate risk-stratification, which again presupposes identification of important risk factors [5, 6]. Risk factors of readmission for patients hospitalized for CAP have not been well described, and previous research has primarily evaluated factors which either pre-exist the pneumonia or reflect the severity at the day of admission [7–12]. Factors, which reflect the cause of hospitalization and the health status at discharge, might, however, be important predictors of readmission [13]. We hypothesized that six different blood-based biomarkers routinely measured in patients with CAP would be associated with risk of readmission after hospitalization for CAP and could be used in a risk-stratification tool.

Thus, the objective of this study was to investigate whether hemoglobin, white blood cell count (WBC), urea, sodium, albumin, and C-reactive protein at discharge in patients hospitalized for CAP are associated with 30-day readmission.

## Methods

### Setting and patients

This study was based on the retrospective *CAP-NORTH Cohort*, which has previously been described in detail by *Egelund* et al. [14]. In brief, the *CAP-NORTH Cohort* was created by evaluating all consecutive patients with an ICD-10 code of pneumonia admitted to three Danish hospitals between January 1, 2011, and July 1, 2012. The inclusion criteria were a new pulmonary infiltrate on chest radiography in combination with at least one symptom of lower respiratory tract infection at admission. Patients with HIV, active tuberculosis, or pharmacological immunosuppression or patients who had been hospitalized within the preceding 28 days of admission were excluded. Patients with more than one admission during the study period were included with reference to the first contact.

In this study, patients who died during the index-hospitalization or had missing data on follow-up or biomarkers at discharge were excluded.

### Variables

Information on demographic characteristics, co-morbidities, initial severity of the pneumonia, and the course of index-hospitalization was collected from medical records. The following co-morbidities were registered: chronic obstructive respiratory disease (COPD), other chronic respiratory diseases, malignancy, chronic heart failure, other chronic heart diseases, neurovascular diseases, other chronic neurological diseases, chronic renal failure, chronic liver failure, and diabetes. The combined burden of co-morbidities was assessed by categorizing patients into three groups based on the sum of conditions (none, one, and more than one). The severity of the pneumonia was measured by the CURB-65 score, which is a composite risk-stratification tool that predicts 30-day mortality in patients with CAP [15].

Biomarkers at discharge were defined as the last available measurement before discharge during the index-hospitalization. In cases with more than one measurement on the same day, the highest value was used. Collection and analysis of blood samples was part of the in-hospital routine management of patients.

Variables that were not mentioned in medical records were recorded as missing. All data were entered into the CAPNETZ database (www.capnetz.de) or into a database programmed in EpiData entry 3.1 (www.epidata.dk). Both databases had error-detection features.

### Outcomes

The outcome measure was all-cause, unplanned readmission within 30 days after discharge from the index-hospitalization. The days from discharge to readmission and the cause of readmission were registered. Further, it was registered if patients died in the follow-up period without a preceding readmission. Patients with multiple readmissions were registered with reference to the first readmission. Data on outcome were collected from medical records, which via linkages to *The Danish National Patient Register* and *The Danish Civil Registration System* hold information on all public hospitalizations and mortality [16].

### Statistical analyses

As all continuous variables were skewed, median and interquartile range (IQR) was reported. Differences in median values were tested by the non-parametric Wilcoxon rank-sum test. Counts and percentages were reported for categorical variables. Differences between categorical variables were tested with Person’s test or Fisher’s exact test, as appropriate.

To account for competing risk in form of death without a preceding readmission, a regression model proposed by *Fine and Gray* [17] was used to calculate sub-distributed hazard ratios for variables based on the cumulative incidence function of readmissions. Data was censored at 30 days after discharge. In the regression analyses, biomarkers were evaluated as binary variables: quartiles for biomarker concentrations were calculated, and for WBC, urea, and C-reactive protein, the upper quartile was used as cut-off, while the lower quartile was used as cut-off for hemoglobin, sodium, and albumin. Variables with *p* < 0.1 in the univariate analyses were included in the multivariate analysis. A risk-stratification tool was derived by dividing patients into groups based on the summarized number of independent risk factors of readmission. Corresponding cumulative incidence curves and hazard ratios were generated.

Two sensitivity analyses were performed: In the first sensitivity analysis, the variable assessing the combined burden of co-morbidities was replaced with individual co-morbidities in the multivariate analysis. In the second sensitivity analysis, the multivariate analysis was restricted to patients, who had a valid measurement of biomarkers within 3 days of discharge.

All *p* values were two-sided and significance levels were < 0.05. All analyses were performed in SAS Enterprise Guide 7.1.

#### Data availability

The datasets generated and analyzed during the current study are available from the corresponding author on reasonable request.

## Results

### Study population

**Fig. 1:**
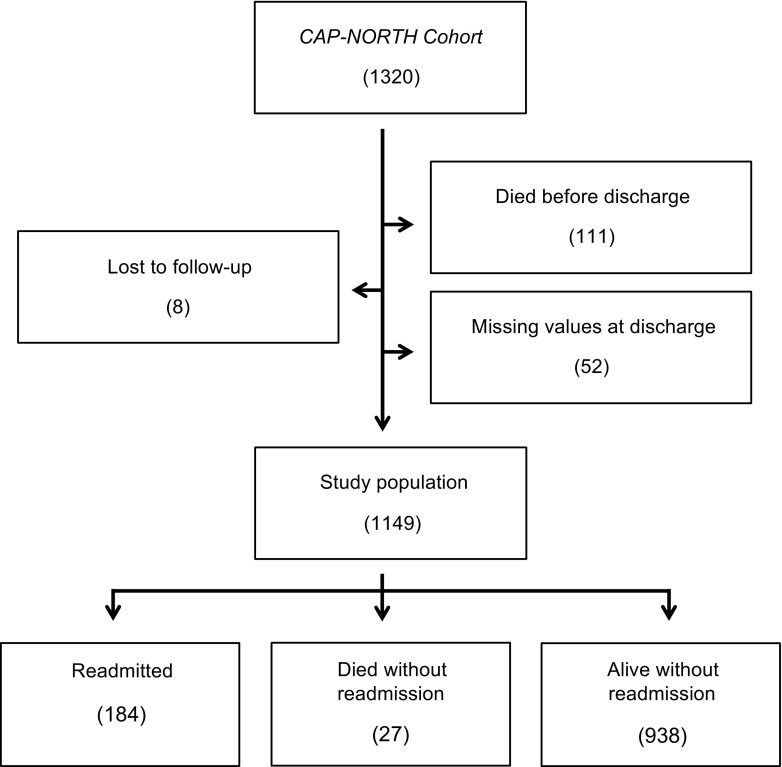
Flow chart of the study population

Of the 1320 patients in the *CAP-NORTH Cohort*, 111 (8.4%) died during the initial hospitalization, 8 (0.6%) were lost to follow-up, and 52 (3.9%) had missing data on biomarkers at discharge (Fig. 1) [12]. These patients were excluded. Thus, the total study population consisted of 1149 patients, who were discharged alive. Patients who were excluded due to missing data on biomarkers were hospitalized and received intravenous (I.V.) antibiotics for a shorter period than the study population (median length of I.V. antibiotic treatment 1 day (IQR, 0–3) vs. 3 days (IQR, 2–6), *p* < 0.001 and median length of stay 1.5 days (IQR, 1–4.5) vs. 5 days (IQR, 3–9), *p* < 0.001). No other differences in baseline characteristics or outcomes were observed (Appendix, Table 1).

### Baseline characteristics

The median age of the study population was 70 years (IQR, 57–81), 40.6% had no record of co-morbidities, 35.7% had 1 co-morbidity, and 23.7% had ≥ 2 (Table 1). Based on the CURB-65 score, 15.7% of the patients had a severe pneumonia at admission (more than 2 points). The median length of stay of the index-hospitalization was 5 days (IQR, 3–9), and the median length of treatment with I.V. antibiotics was 3 days (IQR, 2–6).

**Table 1:** Baseline characteristics of the study population

Baseline characteristic	Study population (*N* = 1149)
Demographic factor	
Age, year, median (IQR)	70 (57–81)
Sex, male	537 (46.7)
Nursing home residency	110 (9.6)
Active smoker	296 (27.8)
Co-morbidities	
Number co-morbidities	
0	462 (40.6)
1	407 (35.7)
≥ 2	270 (23.7)
Malignancy	99 (8.6)
COPD	208 (18.3)
Other chronic respiratory disease	138 (12.1)
Chronic heart failure	90 (7.9)
Other chronic heart disease	174 (15.2)
Chronic liver disease	9 (0.8)
Chronic kidney disease	39 (3.4)
Cerebrovascular disease	109 (9.5)
Other chronic neurological disease	44 (3.8)
Diabetes mellitus	142 (12.2)
Severity of pneumonia at admission	
CURB-65 score	
0–1	559 (55.9)
2	284 (28.4)
3–5	157 (15.7)
Course of hospitalization	
Length of I.V. antibiotics, d, median (IQR)	3 (2–6)
Length of stay, day, median (IQR)	5 (3–9)
ICU admittance	80 (7.0)

Other chronic respiratory diseases were asthma, bronchiectasis, pulmonary fibrosis, sarcoidosis, sleep apnea. Chronic cardiac diseases were cardiac arrhythmia, ischemic heart disease, and cardiomyopathy. Other chronic neurological diseases were epilepsy, Parkinson’s disease, multiple sclerosis and amyotrophic lateral sclerosis. All variables had less than 1% missing values except CURB-65 score (13.0%) and active smoker (7.3%). All variables are reported as numbers and percentages unless otherwise stated

*IQR* interquartile range, *COPD* chronic obstructive pulmonary disease, *I.V.* intravenous, *ICU* intensive care unit

Among 952 patients, where a microbiological test was performed, 217 (22.8%) had a pathogen detected (Table 2). The two most common pathogens were *Streptococcus pneumoniae* (*n* = 61, 6.4%) and *Haemophilus influenzae* (*n* = 55, 5.8%).

**Table 2:** Etiology by study population and tested population

Pathogen	Number	Percentage of study population (*N* = 1149)	Percentage of tested population (*N* = 952)
*Streptococcus pneumoniae*	61	5.3	6.4
*Haemophilus influenzae*	55	4.8	5.8
*Mycoplasma pneumoniae*	35	3.0	3.7
*Moraxella catarrhalis*	19	1.7	2.0
*Staphylococcus aureus*	8	0.7	0.8
*Pseudomonas aeruginosa*	11	1.0	1.2
*Escherichia coli*	8	0.7	0.8
*Legionella pneumophila*	5	0.4	0.5
Others	15	1.3	1.6
Total	217	18.9	22.8

### Outcomes

One hundred eighty-four (16.0%) patients were readmitted within 30 days after discharge and 27 (2.3%) died without a preceding readmission. A total of 56 (4.9%) patients died within the 30-day follow-up period. Pneumonia was the most common cause of readmission accounting for 35.2% of all readmissions (Table 3). Other frequent causes of readmission were pulmonary and cardiovascular diseases, which accounted for 12.5 and 12.0%, respectively.

**Table 3:** Causes of readmission

Cause of readmission	Number (%)
*Pneumonia*	65 (35.2)
*Pulmonary*	23 (12.5)
*Cardiovascular*	22 (12.0)
*Infection*	20 (10.9)
*Malignant*	11 (6.0)
*Surgical*	8 (4.3)
*Neurological*	6 (3.3)
*Other*	29 (15.8)
Total	184 (100)

### Biomarker concentrations at discharge

Patients who were readmitted had higher median WBC concentrations (9.4 vs 8.4 cells × 10^9^/L, *p* < 0.001) and median urea concentrations (5.4 vs. 4.6 mmol/L, *p* = 0.001) and lower median albumin concentrations (35 vs 36 g/L, *p* < 0.001) than patients who were not readmitted (Table 4). Patients were divided into binary groups based on biomarker concentrations, with cut-offs corresponding to the upper quartile for WBC, urea, and C-reactive protein and the lower quartile for hemoglobin, sodium, and albumin. Quartile-based cut-offs were hemoglobin < 7.0 mmol/L, WBC ≥ 10.6 cells × 10^9^/L, urea ≥ 7.0 mmol/L, sodium < 137 mmol/L, albumin < 32 g/L, and CRP ≥ 70 mg/L.

**Table 4:** Biomarker concentrations at discharge

Biomarker at discharge	All (*N* = 1149)	Not readmitted^a^ (*N* = 938)	Readmitted (*N* = 184)	*p* value
Hemoglobin, mmol/L	7.7 (7.0–8.3)	7.7 (7.0–8.3)	7.6 (7.0–8.3)	0.324
WBC, cells × 10^9^/L	8.6 (6.9–10.6)	8.4 (6.7–10.4)	9.4 (7.7–11.8)	< 0.001
Urea, mmol/L	4.7 (3.5–7.0)	4.6 (3.4–6.6)	5.4 (3.7–8.3)	0.001
Sodium, mmol/L	139 (137–141)	139 (137–141)	139 (136–141)	0.799
Albumin, g/L	35 (32–39)	36 (32–39)	35 (30–38)	< 0.001
C-reactive protein, mg/L	38 (18–70)	38 (18–69)	39 (18–72)	0.973

All variables are reported as medians and interquartile ranges. *p* values refer to differences between *not readmitted* and *readmitted* patients

*mmol/L* millimole per liter, *WBC* white blood cell count, *g/L* grams per liter, *mg/L* milligrams per liter

^a^The 27 patients who died without a preceding readmission are not included in the group of patients *not readmitted*

### Risk factors of readmission

**Fig. 2:**
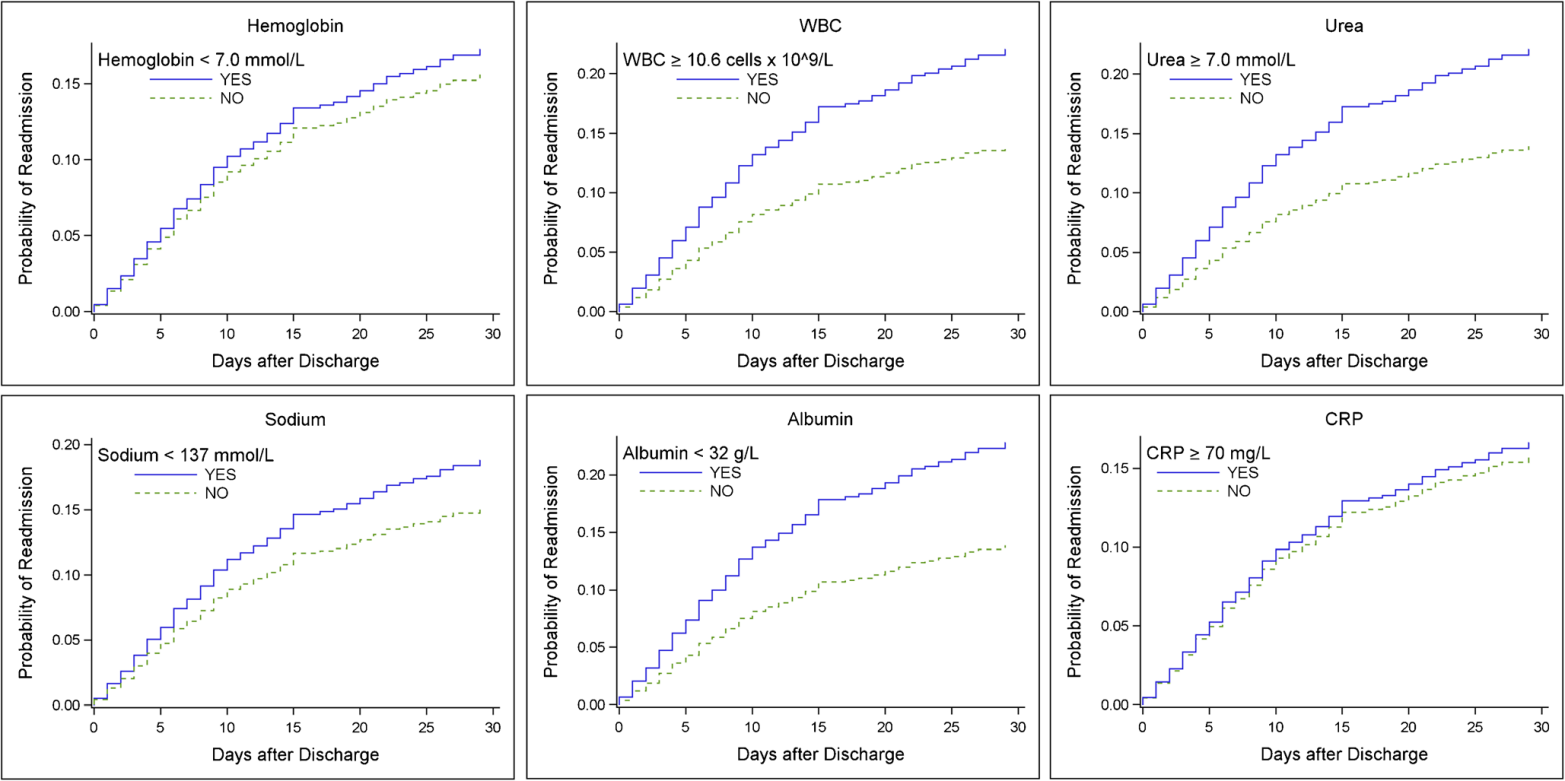
Cumulative incidence curves by biomarker at discharge. Abbreviations: WBC white blood cells, CRP C-reactive protein

In the univariate analyses WBC, urea, and albumin were significantly associated with readmission (Table 5). Hazard ratios were 1.67; 95% CI, 1.23–2.25 for patients with WBC ≥ 10.6 cells × 10^9^/L or urea ≥ 7.0 mmol/L. For patients with albumin < 32 g/L, the hazard ratio was 1.74; 95% CI, 1.28–2.36. Cumulative incidence curves of biomarkers are plotted in Fig. 2.

**Table 5:** Risk factors of readmission

Variable	Unadjusted hazard ratios (95% Cl)	*p* value	Adjusted hazard ratios* (95% Cl)	*p* value
Biomarker at discharge				
Hemoglobin, < 7.0 mmol/L	1.12 (0.80–1.56)	0.511	–	–
WBC, ≥ 10.6 cells × 10^9^/L	1.67 (1.23–2.25)	< 0.001	1.50 (1.07–2.11)	0.018
Urea, > 7.0 mmol/L	1.67 (1.23–2.25)	< 0.001	1.09 (0.75–1.59)	0.660
Sodium, < 137 mmol/L	1.28 (0.93–1.76)	0.137	–	–
Albumin, < 32 g/L	1.74 (1.28–2.36)	< 0.001	1.78 (1.24–2.54)	0.002
C-reactive protein, ≥ 70 mg/L	1.06 (0.76–1.48)	0.717	–	–
Baseline characteristic				
Age, per year	1.02 (1.01–1.03)	< 0.001	1.01 (1.00–1.02)	0.128
Sex, male	1.40 (1.05–1.87)	0.022	1.28 (0.93–1.77)	0.135
Nursing home residency	1.55 (1.02–2.34)	0.041	1.27 (0.76–2.14)	0.360
Active smoker	0.96 (0.69–1.33)	0.794	–	–
Number co-morbidities				
0, *reference*				
1	1.12 (0.78–1.61)	0.549	1.03 (0.69–1.56)	0.875
≥ 2	2.09 (1.48–2.96)	< 0.001	1.74 (1.15–2.64)	0.009
CURB-65 score				
0–1, *reference*				
2	2.27 (1.60–3.23)	< 0.001	1.44 (0.94–2.20)	0.091
3–5	2.20 (1.44–3.36)	< 0.001	1.22 (0.72–2.06)	0.452
Length of I.V. antibiotics, per day	1.03 (1.00–1.05)	0.026	0.99 (0.94–1.04)	0.661
Length of stay, per day	1.02 (1.01–1.03)	0.005	1.02 (0.99–1.04)	0.350
ICU admittance	0.68 (0.35–1.34)	0.266	–	–

*mmol/L* millimole per liter, *WBC* white blood cell count, *g/L* grams per liter, *mg/L* milligrams per liter, *I.V.* intravenous, *ICU* intensive care unit

^a^Due to missing values, a total of 938 patients were used in the multivariate model

In the multivariate analysis, the associations remained significant for WBC and albumin with hazard ratios of 1.50; 95% CI, 1.07–2.11 and 1.78; 95% CI, 1.24–2.54, respectively (Table 5). The only baseline characteristic that was independently associated with readmissions was the combined burden of co-morbidities. The hazard ratio was 1.74; 95% CI, 1.15–2.64 for patients with ≥ 2 co-morbidities compared to patients without co-morbidities.

**Fig. 3:**
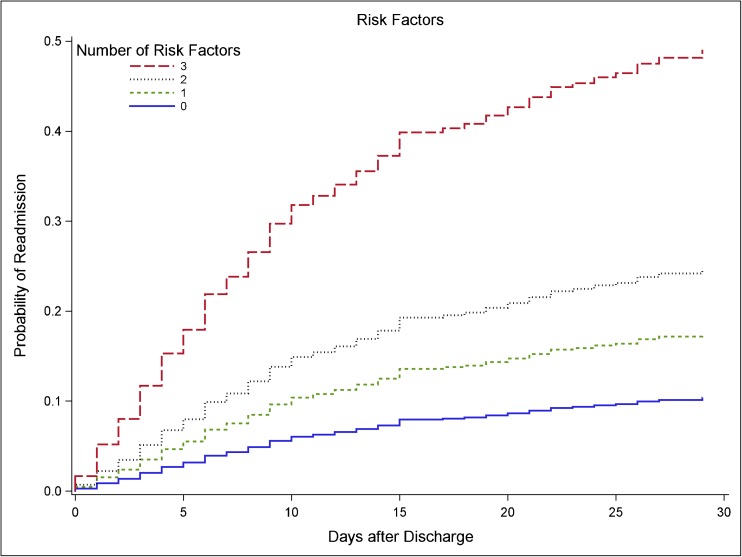
Cumulative incidence curve by number of risk factors. Risk factors were WBC ≥ 10.6 cells × 10^9^/L and albumin < 32 g/L at discharge and the presences of ≥ 2 co-morbidities. Due to missing values, a total of 1139 patients were used in the analysis

In order to identify a combined risk-stratification tool, we assessed the combined risk of all independent risk factors. Figure 3 shows the cumulative incidence curves of 0, 1, 2, and 3 risk factors. The corresponding hazard ratios, where patients without risk factors served as the reference group (*n* = 509), were 1.76, 95% CI, 1.25–2.49 for patients with 1 risk factor (*n* = 454); 2.59, 95% CI, 1.71–3.93 for patients with 2 risk factors (*n* = 149); and 6.15, 95% CI, 3.33–11.38 for patients with 3 risk factors (*n* = 27). As 10 patients had missing data on co-morbidities, the total number of patients in the four groups summarizes to 1139.

### Sensitivity analyses

Replacing the variable assessing the combined burden of co-morbidities with individual co-morbidities in the multivariate model did not change the associations between WBC (hazard ratio 1.53; 95% CI, 1.09–2.16) and albumin (hazard ratio 1.82; 95% CI, 1.28–2.60) and readmission (Appendix, Table 2). Of note, COPD and other neurological diseases were significantly associated with readmission (hazard ratios 1.61; 95% CI, 1.12–2.30 and 2.69; 95% CI, 1.49–4.85, respectively).

The percentages of patients with a measurement of hemoglobin, WBC, urea, sodium, albumin, and C-reactive protein within 3 days before discharge were 86.2, 93.6, 85.0, 88.1, 69.7, and 93.4, respectively. No changes of hazard ratios of WBC (1.78; 95% CI, 1.17–2.72) or albumin (1.87; 95% CI, 1.17–2.98) were observed when the multivariate analysis was restricted to the subset of patients (*n* = 754, 65.6%), where all biomarkers were measured within 3 days before discharge (Appendix, Table 3).

## Discussion

In this retrospective, multi-center, cohort study of 1149 patients discharged after hospitalization for CAP, we found that WBC ≥ 10.6 cells × 10^9^/L and albumin < 32 g/L at discharge and the presence of ≥ 2 co-morbidities are independent risk factors of 30-day readmission. Further, when WBC, albumin, and co-morbidities were combined into a risk-stratification tool, we found a step-wise increase in risk of readmission according to the number of risk factors.

Previous research on identification of risk factors of readmission after hospitalization for CAP is limited and factors which primarily have been evaluated either pre-exist the pneumonia or reflect the severity at the day of admission. Apart from the presence of co-morbidities, which throughout the literature are found to be an independent risk factor readmission, other frequently evaluated factors, such demographic characteristics and the initial severity of the pneumonia, are not consistently found to be associated with readmission [7–12]. To the best of our knowledge, this study is the first to both evaluate and demonstrate associations between albumin and WBC at discharge and readmission after CAP. Biomarkers at discharge may reflect the health status of patients during a period of recovery and could provide additional information on the risk of readmission besides what can be obtained at the day of admission. Correspondingly, some studies in different patient populations, have found that risk-stratification tools of readmission are improved when data from the entire hospital stay are added to data from the first day of hospitalization [18, 19]. Length of hospitalization, duration of antibiotic treatment, and signs of clinical instability at discharge, which also relate to the health status beyond the day of admission, are, however, not consistently found to be predictive of readmissions after hospitalization for CAP [7–12].

As elevated WBC and depressed albumin are common findings during acute infections, our results could reflect that some patients were discharged too early from the hospital with an on-going pneumonia. Unfortunately, clinical parameters beyond the day of admission were not available in the *CAP-NORTH Cohort*, and thus, we could not review whether these patients also had clinical signs of active infection at discharge. There are, however, some indications that treatment and discharge patterns in our study were not different from other studies. First, with a median and IQR of WBC at discharge of 8.6 and 6.9–10.6 cells × 10^9^/L, results from our study was very similar to results from another study of patients with CAP, which reported a median and IQR of WBC at discharge of 8.3 and 6.5–11.0 cells × 10^9^/L. Second, in terms of treatment, both the length of hospitalization and the duration of I.V. antibiotic therapy in our study were within international recommendations for treatment of CAP [20]. And third, the outcome in our study, both as the overall readmission rate and the proportion of readmissions caused by pneumonia, were similar to reports from other studies, which would not be expected if patients were discharged too early with an on-going pneumonia [7–12, 21].

Elevated WBC at discharge could also reflect persistent inflammation rather than active infection*.* In a study from 2008, *Yende* et al. [22] reported that high discharge concentrations of the pro-inflammatory marker IL-6 were associated with an increased risk of mortality 3 months after hospitalization for CAP. As the authors also suggested, one possible mechanism could be that a persistent inflammation after acute infections increases the risk of cardiovascular events in the same way as elevated levels of inflammatory markers increase the risk in other populations [23].

In 2013, *Krumholz* [24] proposed that stressors occurring during hospitalization, such as malnutrition, sleep deprivation, and immobility, increase the vulnerability of patients after discharge, a phenomenon described as a *post-hospital syndrome.* These factors, and not only pre-existing conditions or the severity of the disease at the day of admission, may significantly affect the risk of readmission. Biomarkers measured at discharge might pose a simple and reliable way to assess some of the impact that hospitalization has on the health status of the patients, and thus be useful in identification of patients in high risk of readmission.

When WBC, albumin, and co-morbidities are combined into a risk-stratification tool, the study population could be divided into four groups with a step-wise increase in risk of readmission. This suggests that the combination of biomarkers at discharge and co-morbidities could be used to identify patients which are in need of post-discharge interventions. There are, however, need for validation in prospective studies.

### Limitations

Our study had some limitations. First, due to the retrospective design of the study, the measurement of biomarkers was part of the routine management of patients. This could have led to a selection bias, as 52 patients was excluded due to missing values, and thus, our findings may not be generalized to all patients hospitalized for CAP. The 52 patients who were excluded were hospitalized and received I.V. antibiotics for a shorter period than the study population, but otherwise, no differences in either baseline characteristics or outcomes were observed. Further, in a subset of patients, the last measurement of some biomarkers was performed several days before discharge, which could limit the reproducibility of our results. No changes in associations were, however, observed when the statistical analysis was restricted to patients who had all biomarkers measured within 3 days of discharge. Second, as we were limited to data which could be obtained from patient records, we could not evaluate the effect of some potential risk factors of readmission, including alcohol abuse, clinical stability on discharge, and discharge dispositions. Such factors could potentially confound our results. Third, we cannot account for inappropriate antibiotic treatment, which could be a risk factor for readmission. However, to our knowledge, no previous studies have found that inappropriate antibiotic treatment is associated with increased risk of readmission after CAP. Fourth, this cohort was based on patients hospitalized between January 2011 and July 2012, and both demographic characteristics and guidelines of treatment of pneumonia could have changed since then, which might limit the generalizability of our results.

### Strengths

The main strength of this study was that we were able to include all consecutive patients with x-ray-confirmed CAP for a full 18 months, thus reducing the risk of inclusion bias. Further, this study was multi-center, and the three hospitals from where patients were included supported an entire region in Denmark. We also had robust and almost complete data regarding death, readmission, and causes of readmission, due to access *The Danish National Patient Register* and *The Danish Civil Registration System.*

## Conclusions

In this study, we showed that discharge concentrations of WBC ≥ 10.6 cells × 10^9^/L and albumin < 32 g/L and the presences of ≥ 2 co-morbidities were associated with an increase in risk of 30-day readmission for patients hospitalized for CAP. The combination of biomarkers at discharge and co-morbidities could be used to stratify patients according to risk of readmission, and thus help guide post-discharge interventions. However, due to the retrospective design of this study, our results should be validated.

## Electronic supplementary material

ESM 1 (DOCX 18 kb)
