# Supplementary material for: Associations between biomarkers at discharge and co-morbidities and risk of readmission after community-acquired pneumonia: a retrospective cohort study
Source: Eur J Clin Microbiol Infect Dis. 2018 Mar 29;37(6):1103–11. doi: 10.1007/s10096-018-3224-8 (PMC5948264; doi:10.1007/s10096-018-3224-8)
Supplement: Supplementary file 1 — (DOCX 18 kb) [file 10096_2018_3224_MOESM1_ESM.docx]

APPENDIX (electronic supplementary material)

Table 1 in appendix

Comparison between patient with missing biomarkers and patients with all biomarkers

| **Variables** | **Patients with**  **missing biomarkers**  **(n = 52)** | **Patients with**  **all biomarkers**  **(n = 1149)** | ***P* value** |
| --- | --- | --- | --- |
| Demographic factor |  |  |  |
| Age, y, median (IQR) | 68 (50-77) | 70 (57-81) | 0.106 |
| Sex, male | 21 (40.38) | 537 (46.74) | 0.369 |
| Nursing home residency | 2 (3.85) | 110 (9.61) | 0.163 |
| Active smoker | 9 (19.57) | 296 (27.79) | 0.221 |
| Comorbid condition |  |  |  |
| Number of comorbidities |  |  |  |
| 0 | 25 (48.08) | 462 (40.56) | 0.125 |
| 1 | 21 (40.38) | 407 (35.73) |  |
| ≥ 2 | 6 (11.54) | 270 (23.71) |  |
| Severity of pneumonia at admission |  |  |  |
| CURB-65 score |  |  |  |
| 0-1 | 26 (63.41) | 559 (55.90) | 0.628 |
| 2 | 10 (24.39) | 284 (28.40) |  |
| 3-5 | 5 (12.20) | 157 (15.70) |  |
| Course of hospitalization |  |  |  |
| Length of I.V. antibiotics, d, median (IQR) | 1 (0-3) | 3 (2-6) | < 0.001 |
| Length of stay, d, median (IQR) | 1.5 (1-4.5) | 5 (3-9) | < 0.001 |
| Outcome |  |  |  |
| Readmitted | 11 (21.15) | 184 (16.01) | 0.611 |
| Dead without readmission | 1 (1.92) | 27 (2.35) |  |
| Alive without readmission | 40 (76.92) | 938 (81.64) |  |
| Abbreviations: y, years; IQR, interquartile range; I.V., intravenous: d, days.  All variables are reported as numbers and percentages unless otherwise stated. | | | |

Table 2 in appendix

Sensitivity analysis: Evaluation of individual co-morbidities in the multivariate model

| **Variable** | **Adjusted Hazard Ratios* (95% Cl)** | ***P* value** |
| --- | --- | --- |
| Biomarker at discharge |  |  |
| WBC, ≥ 10.6 cells x 10^9^/L | 1.53 (1.09-2.16) | 0.015 |
| Urea, > 7.0 mmol/L | 1.16 (0.79-1.69) | 0.457 |
| Albumin, < 32 g/L | 1.82 (1.28-2.60) | 0.001 |
|  |  |  |
| Baseline characteristic |  |  |
| Age, per y | 1.01 (1.00-1.02) | 0.095 |
| Sex, male | 1.34 (0.98-1.84) | 0.070 |
| Nursing home residency | 1.17 (0.68-2.02) | 0.578 |
| Co-morbidities |  |  |
| Malignancy | 0.96 (0.54-1.71) | 0.901 |
| COPD | 1.61 (1.12-2.30) | 0.010 |
| Other chronic respiratory disease | 1.26 (0.78-2.05) | 0.346 |
| Chronic heart failure | 1.05 (0.64-1.72) | 0.855 |
| Other chronic heart disease | 1.21 (0.80-1.84) | 0.366 |
| Chronic liver disease | 1.27 (0.33-4.89) | 0.728 |
| Chronic kidney disease | 1.63 (0.87-3.07) | 0.127 |
| Cerebrovascular disease | 1.09 (0.64-1.87) | 0.752 |
| Other chronic neurological disease | 2.69 (1.49-4.85) | 0.001 |
| Diabetes mellitus | 0.92 (0.57-1.47) | 0.715 |
| CURB-65 score |  |  |
| 0-1, *reference* |  |  |
| 2 | 1.41 (0.92-2.17) | 0.115 |
| 3-5 | 1.16 (0.69-1.97) | 0.578 |
| Length of I.V. antibiotics, per d | 0.99 (0.95-1.04) | 0.763 |
| Length of stay, per d | 1.01 (0.98-1.04) | 0.415 |
| Abbreviations: WBC, white blood cell count; mmol/L, millimoles per Liter; g/L, grams per Liter; y, year; COPD, chronic obstructive pulmonary disease; I.V., intravenous; d, day.  *Due to missing values a total of 938 patients were used in the multivariate model. | | |

Table 3 in appendix

Sensitivity analysis: Restriction of the multivariate model to patients with all biomarkers measured within 3 days of discharge

| **Variable** | **Adjusted Hazard Ratios*** **(95% Cl)** | ***P* value** |
| --- | --- | --- |
| Biomarker at discharge (≤ 3 days)† |  |  |
| WBC, ≥ 10.6 cells x 10^9^/L | 1.78 (1.17-2.72) | 0.008 |
| Urea, > 7.0 mmol/L | 0.84 (0.51-1.39) | 0.497 |
| Albumin, < 32 g/L | 1.87 (1.17-2.98) | 0.009 |
|  |  |  |
| Baseline characteristic |  |  |
| Age, per y | 1.01 (0.99-1.02) | 0.561 |
| Gender, male | 1.13 (0.75-1.72) | 0.552 |
| Nursing home residency | 1.23 (0.61-2.49) | 0.562 |
| Number of co-morbidities |  |  |
| 0, *reference* |  |  |
| 1 | 1.23 (0.73-2.08) | 0.439 |
| ≥ 2 | 2.27 (1.35-3.84) | 0.002 |
| CURB-65 score |  |  |
| 0-1, *reference* |  |  |
| 2 | 1.98 (1.21-3.25) | 0.007 |
| 3-5 | 0.83 (0.40-1.73) | 0.612 |
| Length of I.V. antibiotics, per d | 1.03 (0.97-1.09) | 0.295 |
| Length of stay, per d | 1.00 (0.95-1.04) | 0.891 |
| Abbreviations: WBC, white blood cell count; mmol/L, millimoles per Liter; g/L, grams per Liter; y, year; I.V., intravenous; d, days.  * Due to missing values a total of 659 patients were used in the multivariate model.  †A total of 754 patients (65.6 %) had all biomarkers measured within 3 days of discharge. | | |
